# Antimicrobial Resistance in Poultry Farming: A Look Back at Environmental *Escherichia coli* Isolated from Poultry Farms during the Growing and Resting Periods

**DOI:** 10.1155/2023/8354235

**Published:** 2023-11-28

**Authors:** Sukanya Thongratsakul, Patamabhorn Amavisit, Chaithep Poolkhet

**Affiliations:** ^1^Department of Veterinary Public Health, Faculty of Veterinary Medicine, Kasetsart University, Kamphaeng Saen Campus, Nakhon Pathom 73140, Thailand; ^2^Department of Microbiology and Immunology, Faculty of Veterinary Medicine, Kasetsart University, Bangkok 10900, Thailand

## Abstract

During the production cycle of poultry farms, pathogens may remain in the next cycle of rearing young chickens. This study was conducted at three industrial chicken farms (A, B, and C) in central Thailand. Results showed that the percentages of *E. coli* during the resting period in farms A, B, and C were 28.6, 53.8, and 7.8, respectively, and those during the growing period were 45, 68.8, and 75. The most common resistant patterns during the resting period in all farms were AML-AMP-SXT and AML-AMP-DO-SXT, and those during the growing period were AML-AMP and AML-AMP-SXT. The locations of *bla*_TEM_-positive *E. coli* isolates from the inside houses (inside buildings) of all farms included cloacal swabs, floors, water nipples, pan feeders, and husks, whereas that from the outside environment included boots, wastewater, soil, and water from cooling pads and tanks. Our results indicate that the percentage of antimicrobial resistance (AMR) and its pattern depend on the husbandry period and the strictness of biosecurity. Moreover, our findings derived from samples gathered from broiler farms between 2013 and 2015 align with those of the current studies, highlighting persistent trends in *E. coli* resistance to various antimicrobial agents. Therefore, enhancing biosecurity measures throughout both the resting and growing periods is crucial, with a specific focus on managing raw materials, bedding, breeding equipment, and staff hygiene to reduce the transmission of antimicrobial resistance in poultry farms.

## 1. Introduction

The broiler industry is currently developing breeding and production systems to maximize productivity and efficiency. With a focus on food safety, in accordance with Good Agricultural Practice (GAP) standards, producers have sought to establish steps to reduce bacterial contamination, particularly in intensive farming systems. Production systems may require antimicrobials for prophylaxis for disease control and to reduce the incidence of pathogenic infections in poultry farms [1]. Consequently, microorganisms may acquire antimicrobial resistance.

Antimicrobial-resistant bacteria may transmit their genetic traits to the environment, and pathogenic bacteria circulate within the farm. Therefore, the future use of antimicrobials for disease control in poultry farms may be ineffective. Furthermore, antimicrobial-resistant bacteria can contaminate food products related to poultry; thus, consumers may be exposed to these dangerous bacteria [2]. Many studies have investigated antimicrobial-resistant bacteria using *Escherichia coli*, which can be a reservoir of resistance genes that can be transferred horizontally to other bacteria, such as antimicrobial resistance (AMR) sentinels [3, 4].

Previous studies have indicated that animal manure can be a reservoir of resistance genes. Owing to its use as soil fertilizer, animal manure can also disseminate AMR genes, a process that affects the distribution of resistant bacteria during production periods in poultry farms. Insufficient sanitation during the previous production cycles may lead to an increased abundance of resistant bacteria, which may result in environmental contamination and gene transfer to humans [5, 6].

AMR sentinels are useful for studying AMR background data and the proliferation of residual resistance genes on farms. The distribution of resistance genes during the growing period reflects the spread of residual resistance genes from the previous breeding cycle due to insufficient sanitation. Moreover, it also reflects how resistance genes can become widespread through the introduction of raw materials, equipment, and chickens, which can be AMR reservoirs. Therefore, the aim of this study was to examine AMR in environmental *E. coli* isolated from poultry farms to elucidate the transfer of AMR genes from the environment to animals using a sentinel bacterium.

## 2. Materials and Methods

### 2.1. Ethical Approval

During the data collection period, the investigators diligently acquired verbal informed consent from all farm owners or data providers following the guidelines outlined in the Helsinki Declaration.

### 2.2. Samples and Study Framework

In 2013-2014, swab samples were collected from broiler farms, which are the typical commercial and intensive farms located in central Thailand, for disease monitoring purposes. Farm management is classified into two categories: sector 1 (high level of biosecurity, industrial, and vertically integrated systems) and sector 2 (moderate-to-high level of biosecurity, commercial poultry, and nonvertically integrated systems), according to the FAO classification [7, 8]. Three farms (A, B, and C) were selected for this study. To explore the AMR situation on a farm, commensal *E. coli* was selected as an AMR sentinel, as it can be a reservoir of resistance genes that can spread horizontally to other bacteria [9, 10].

**Figure 1:**
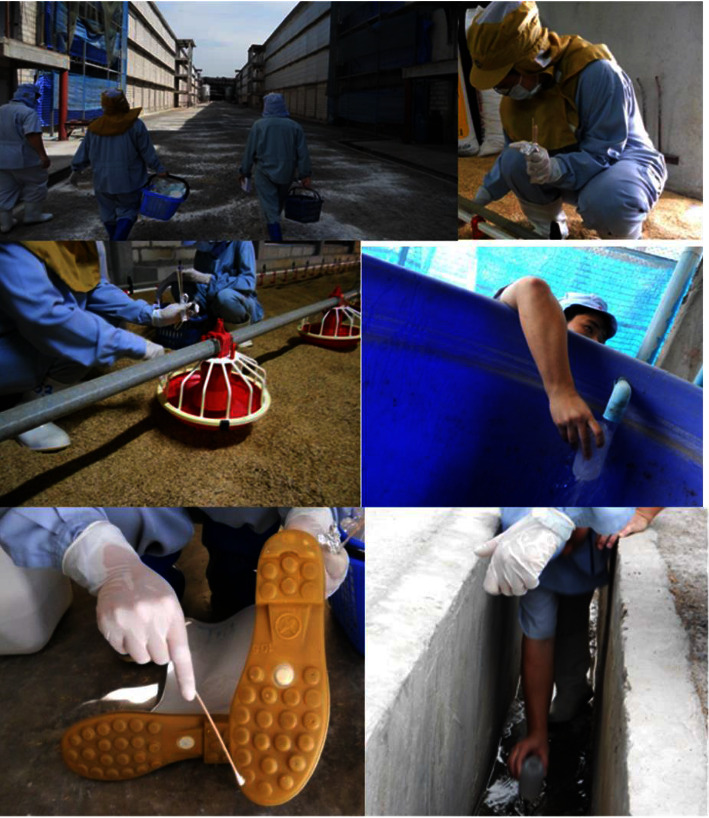
Sample collection at the poultry farm.

Samples were collected during two periods: growing and resting periods. During each period, samples were collected from two houses per farm, and all other houses were considered homogeneous. Specifically, soil, floors, litter, boots, water, and wastewater samples from inside and outside of poultry houses were collected using the swab method (Figure 1). In this study, we collected samples as thoroughly as possible and covered all possible locations and equipment where *E. coli* was most likely to appear. A total of 229 samples were collected from three farms, of which 124 and 105 samples were from the growing and resting periods, respectively.

### 2.3. *E. coli* Identification and Antimicrobial Susceptibility Testing

All samples were submitted for *E. coli* identification at the Diagnostic Unit of the Faculty of Veterinary Medicine, Kasetsart University, Kamphaeng Sean Campus. The isolates were subcultured on MacConkey agar (Oxoid Ltd., UK), and three typical colonies of *E. coli* were chosen for species confirmation using biochemical testing based on IMVics (indole, methyl red, Voges–Proskauer, and Simmons citrate) [11]. *E. coli* isolates were kept in skim milk and stored at −20°C until analyzed.

Antimicrobial susceptibility testing was performed using the agar disk diffusion method, according to the Clinical and Laboratory Standards Institute (CLSI) guidelines [12]. Antimicrobial agents included amoxicillin (AML) 10 *µ*g, amoxicillin clavulanic acid (AMC) 30 *µ*g, ampicillin (AMP) 10 *µ*g, cephalexin (CL) 30 *µ*g, ciprofloxacin (CIP) 30 *µ*g, doxycycline (DO) 30 *µ*g, gentamicin (CN) 10 *µ*g, and sulfamethoxazole–trimethoprim (SXT) 25 *µ*g. *E. coli* (ATCC 25922) was used as control.

### 2.4. Detection of Extended-Spectrum Beta-Lactamase Gene

**Figure 2:**
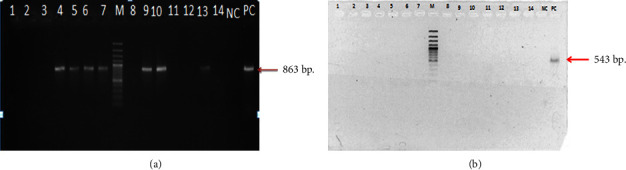
Gel electrophoresis for *bla*_TEM-10_ with positive and negative control (a) and for *bla*_CTX-M_ with positive and negative control (b).

*E. coli* DNA was extracted using the boiling method and used for the amplification of ESBL class A genes, which comprised *bla*_TEM_, *bla*_CTX-M_, and *bla*_SHV_. The *bla*_TEM_, *bla*_CTX-M_, and *bla*_SHV_ were amplified using the primer pairs tem1 FW (ATG AGT ATT CAA CAT TTC CG) and tem2 RW (CTG ACA GTT ACC AAT GCT TA (GenBank accession number U09188); MA1-FW (SCS ATG TGC AGY ACC AGT AA) and MA2-RW (CCG CRA TAT GRT TGG TGG TG) (GenBank accession number X92506); and shv1-FW (GGT TAT GCG TTA TAT TCG CC) and shv2-RW (TTA GCG TTG CCA GTG CTC) (GenBank accession number M59181), respectively. The PCR conditions were as follows: initial denaturation at 98°C for 3 min; followed by 35 cycles of denaturation at 98°C for 30 s, annealing at 55°C for *bla*_TEM_ and *bla*_SHV_, or at 52°C for *bla*_CTX-M_ for 30 s, and extension at 72°C for 35 s, followed by a final extension step at 72°C for 5 min. *K. pneumoniae* (ATCC 700603) was used as an ESBL-positive control (Figure 2).

### 2.5. Statistical Analysis

Descriptive statistics were used to report the presence of antimicrobial susceptibility profiles of *E. coli* strains, as well as that of target genes during the growing and resting periods. A double dendrogram with a clustered heat map (NCSS 2019; NCSS, LLC, Utah, USA) [13] was used to explain the relationship between AMR patterns and antimicrobial susceptibility. Furthermore, the Pearson *χ*2 test was used to determine the statistical difference in the presence of *E. coli* during the growth and resting periods.

## 3. Results

### 3.1. Farm Characteristics

Farms A and C were classified as high biosecurity sector 1. Sanitizers, UV boxes, toilets, cloths, and towels were supplied to each guest before entering the chicken house. The areas surrounding the chicken houses were clean and mostly made of concrete, and some soil was present around the houses. Moreover, in farm C, the areas surrounding the chicken house were clean and orderly, and limestone powder was used as a sanitizer for cleaning the concrete. Meanwhile, Farm B is classified as a moderate biosecurity sector 2. The areas surrounding the chicken house on Farm B were not as clean as those on the other farms, and there was some wastewater around the chicken house. Land water was used as the water supply system for farm B, and the water was treated with chlorine prior to use. A total of 28, 26, and 51 samples from the resting period; and 40, 32, and 52 from the growing period, were collected from Farms A, B, and C, respectively.

### 3.2. *E. coli* Identification and Antimicrobial Susceptibility Results

A total of 105 *E. coli* isolates were obtained from 229 swab samples (Table 1). Further, 28.6%, 53.8%, and 7.8% of samples during the resting period, and 45%, 68.8%, and 75% of samples during the growing period from Farms A, B, and C, respectively, harbored *E. coli*. No significant difference on the number of isolates was observed between the two periods in Farm C (*P* < 0.01) (Table 1).

The percentages of AMR in all farms (A, B, and C) during the resting periods were AML (37.5, 100, 0); AMC (0, 0, 0); AMP (25, 100, 0); CL (0, 0, 0); CIP (0, 37.5, 0), DO (25, 57.2, 25), CN (12.5, 0, 0); and SXT (50, 64.3, 0). Whereas the percentages of AMR in all farms during growing periods were AML (88.9, 95.4, 97.4); AMC (0, 4.5, 0); AMP (83.3, 95.4, 97.4); CL (0, 4.5, 0); CIP (0, 22.7, 7.7); DO (16.7, 31.8, 15.4); CN (0, 27.3, 20.5); and SXT (55.6, 45.4, 33.3) (Table 2). Among all the isolated strains, 76.9% (20/26) and 97.5% (77/79) were resistant to at least one of the eight antimicrobial agents during the resting and growing periods, respectively. Furthermore, the MDR percentage in Farms A, B, and C during the resting periods were 7.7, 34.6, and 0, respectively, while that during the growing periods were 2.5, 8.9, and 7.6, respectively (Tables 2 and 3). Sixteen types of resistance patterns were observed (Table 4), among which AML-AMP-SXT (5/20) and AML-AMP-DO-SXT (5/20) were the most common during the resting period, whereas AML-AMP (23/77) and AML-AMP-SXT (21/77) were the most common during the growing period.

**Figure 3:**
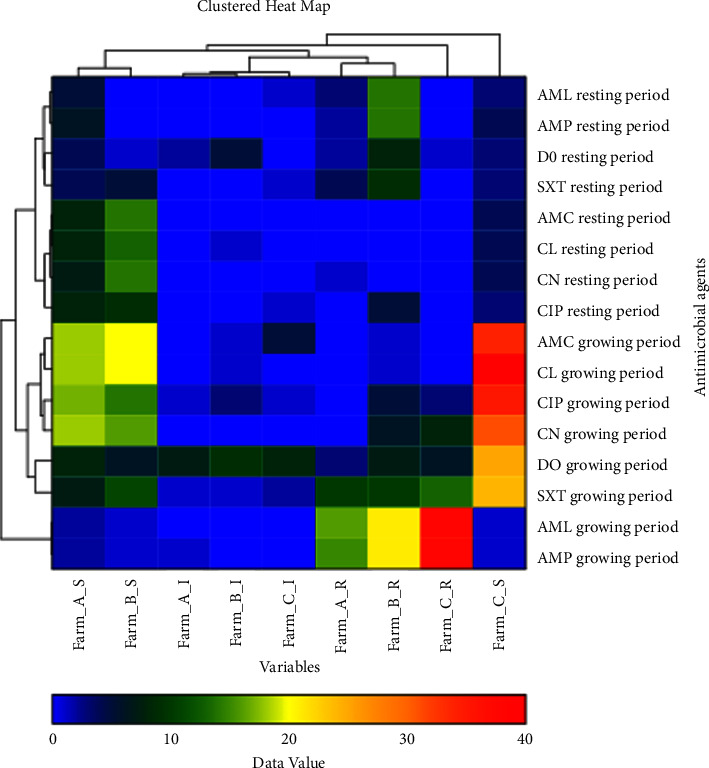
Clustered heat map and double dendrogram of *Escherichia coli.*

Cluster analysis was performed to determine the relationship between AMR patterns and antimicrobial susceptibility of *E. coli* on different farms during the resting and growing periods. The analysis is shown in a double dendrogram, in which the color shades and branches of the dendrogram are observed. Results showed that farms A and B are closely related, whereas farm C had cluster dissimilarity (orange-red color) compared to the other farms as shown in Figure 3.

### 3.3. ESBL Genotypes of *E. coli* Isolates

As a result of the high resistance rate of AML and AMP, three targeted genes (*bla*_TEM_, *bla*_CTX-M_, and *bla*_SHV_) were selected to investigate the present resistance genes. Only *bla*_TEM_ was found in all *E. coli* isolates in this study. *Bla*_TEM_-positive *E. coli* were isolated from cloacal swabs, floors, water nipples, pan feeders, husk and husk housing, boots before and after cleaning, wastewater, soil, and water from cooling pads and tanks (Table 5).

During the resting period in Farms A and C, *bla*_TEM_-positive *E. coli* was detected from wastewater and soil outside poultry houses, indicating that these farms have clean equipment. Meanwhile, in farm B, *bla*_TEM_ was found in both the outside environment and on equipment such as nipples, pan feeders, and floors inside the poultry house. During the growing period, we observed the spread of *bla*_TEM_-positive *E. coli* both inside and outside all poultry houses (Table 5). In addition, a significant (*χ*2 = 19.53, *P* < 0.05, *df* = 1) correlation between the resistance rate of the beta-lactam group and the genotype of *bla*_TEM_-positive *E. coli* was observed.

## 4. Discussion

Sanitation practices follow the standards used for biosecurity management at each farm, including the preparation of poultry houses by cleaning, disinfecting, and killing insects before starting each new crop cycle. The presence of *E. coli* during the resting and growing periods provide insight into the distribution of this organism circulating in the farm. Interestingly, Farm C, which maintained very good disinfection management, had a statistically significant positive *E. coli* rate in both periods. During the growing period, the equipment used to raise poultry in the house, such as nipple waterers and pan feeders, had a high *E. coli* percentage, indicating possible contamination with raw materials. The water supply was not found to have *E. coli-*positive samples, indicating that the water treatment systems on all farms were operational.

An antimicrobial susceptibility test found a high rate of resistance to AMP and AML, which belong to the beta-lactam group. This group is implicated in the prevalence of antimicrobial-resistant bacteria in animal farms [14–16]. Our research findings, derived from samples collected from broiler farms between 2013 and 2015, are consistent with the current work in our field, highlighting the enduring congruence between our observed circumstances and outcomes and the present situation. Notably, our results indicated an increase in the resistance of *E. coli* strains to antimicrobial compounds such as AMP, SXT, and TET. These findings concur with recent research indicating heightened resistance among *E. coli* isolates to beta-lactams, tetracyclines, macrolides, and sulfonamides, further substantiating persistent trends in AMR [17–20]. Most of the resistance patterns were AML-AMP 29.9% (23/77) and AML-AMP-SXT 27.7% (21/77) during the growing period, and AML-AMP-DO-SXT 25% (5/20) and AML-AMP-SXT 25% (5/20) during the resting periods. These types are frequently found in all farms, particularly in wastewater and insects.

According to the cluster analysis results, Farm C exhibited cluster dissimilarity because it had a high degree of biosecurity for controlling and preventing the spread of infections within the farm. Therefore, biosecurity should place more emphasis on waste and pest control systems for animal manure, which could serve as a reservoir for antimicrobial-resistant bacteria [21]. The high percentage of *bla*_TEM_ genes in the environment, waste, and equipment during the growing period indicates that biosecurity may not be sufficient to control AMR. In addition, owing to the high percentage of resistance genes, waste, manure, and drainage systems on farms should be of concern, as these can become AMR reservoirs that transfer resistance genes to the surrounding environment and directly affect poultry health [22, 23].

The current study showed the location of *bla*_TEM_-positive *E. coli* spreading in poultry farms, suggesting that biosecurity measures need to be more focused on poultry production, either during the resting or growing periods. This finding is consistent with those of previous studies that showed a high prevalence of ESBL-producing *E. coli* on poultry farms [24, 25]. AMR genes have been detected in animals and raw materials; therefore, bedding and breeding equipment must be prioritized. Moreover, staff hygiene may play a role, as mechanical transmitters should be monitored to reduce AMR spread on poultry farms.

## 5. Conclusions

In the poultry industry, producers aim to control and prevent pathogen contamination during production by implementing biosecurity programs. Our study findings revealed a substantial prevalence of antimicrobial-resistant *E. coli* during the resting and growing periods, which is consistent with the cluster analysis results. Therefore, stringent biological control measures in raw material management, environmental stewardship, and waste disposal systems should be focused, with the primary objective of mitigating the potential risks associated with pathogen recirculation in subsequent production cycles.

## Figures and Tables

**Table 1:** The number of *Escherichia coli* isolates during the resting and growing periods in three poultry farms.

Bacteria	Farm	Period	Positive/total swabs (%)	*P* value	Pearson *χ*^2^value^1^, *df*^2^
*E. coli* (*n* = 105)	A	Resting	8/28, 28.6	0.17	1.88, 1
	A	Growing	18/40, 45.0		
	B	Resting	14/26, 53.8	0.24	1.35, 1
	B	Growing	22/32, 68.8		
	C	Resting	4/51, 7.8	<0.01	47.75, 1
	C	Growing	39/52, 75.0		

^1^Pearson *χ*^2^ test was used to calculated *P* value of positive and negative samples in different periods of each farm. ^2^*df* = degree of freedom.

**Table 2:** Antimicrobial susceptibility of *E. coli,* categorized as farming periods.

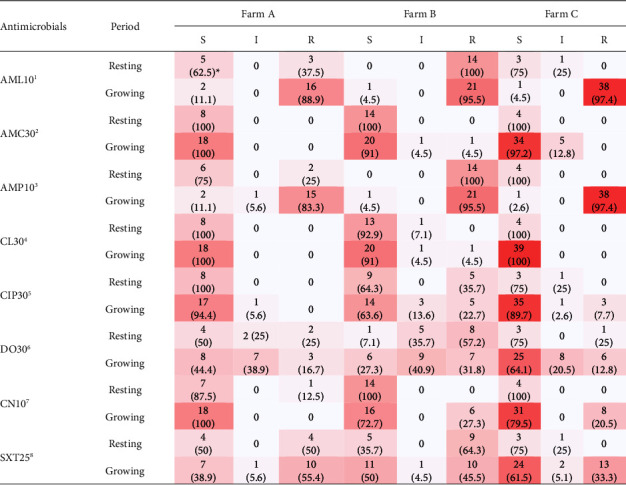

^1^amoxicillin 10 *µ*g, ^2^amoxicillin–clavulanic acid 30 *µ*g, ^3^ampicillin 10 *µ*g, ^4^cephalexin 30 *µ*g, ^5^ciprofloxacin 30 *µ*g, ^6^doxycycline 30 *µ*g, ^7^gentamicin 10 *µ*g, ^8^sulfamethoxazole–trimethoprim (SXT) 25 *µ*g. ^*∗*^Percentage of antimicrobial susceptibility testing of *Escherichia coli* in each farm (Farm A (8, 18), B (14, 22), and C (4, 39)) during the resting and growing periods.

**Table 3:** Resistance patterns of *E. coli* isolated from the three poultry farms.

Patterns of antimicrobial resistance	Farm	Resting period (*n*, %)	Growing period (*n*, %)
None	A	3 (11.5)	0
	B	0	1 (1.3)
	C	3 (11.5)	1 (1.3)

1 type of resistance	A	2 (7.7)	9 (11.4)
	B	1 (3.8)	5 (6.3)
	C	1 (3.8)	14 (17.7)

2 types of resistance	A	1 (3.8)	7 (8.9)
	B	4 (15.4)	9 (11.4)
	C	0	18 (22.8)

≥3 types of resistance	A	2 (7.7)	2 (2.5)
	B	9 (34.6)	7 (8.9)
	C	0	6 (7.6)

Total		26 (100)	79 (100)

**Table 4:** Resistance patterns of *Escherichia coli* isolate from farms A, B, and C.

Antimicrobials\resistance patterns	Resting period (*n* = 20)	Growing period (*n* = 77)
AML	1	—
DO	1	—
SXT	1	2
AML-AMP	1	23
CN-SXT	1	—
AML-AMP-CIP	—	3
AML-AMP-CN	—	4
AML-AMP-DO	—	8
AML-AMP-SXT	5	21
AML-DO-SXT	—	1
AML-AMP-CIP-CN	—	2
AML-AMP-CIP-DO	4	1
AML-AMP-CN-SXT	—	3
AML-AMP-DO-CN	—	3
AML-AMP-DO-SXT	5	2
AML-AMP-CIP-CN-SXT	—	3
AML-AMP-CIP-DO-SXT	1	—
AML-AMP-AMC-CL-DO-CN-SXT	—	1

Total	20	77

**Table 5:** Location and numbers of *bla*_TEM_-positive *E. coli* during the resting and growing periods.

Locations	*bla*_TEM_ positive/number of *E. coli* isolates (%)			
	Resting period	Farm	Growing period	Farm
*Inside the housing*				
Cloacal swabs	0/0	—	9/9 (100)	A, B, C
Floors	3/5 (60)	B	15/18 (83.3)	B, C
Husks	0/0	—	4/5 (80)	A, C
Nipples	1/1 (100)	B	16/16 (100)	A, B, C
Pan feeders	1/1 (100)	B	13/14 (92.8)	B, C

*Outside the housing*				
Insects	0/1	—	1/1	A
Pipe of wastewater	0/1	A	0/0	—
Boots	0/1	—	7/7 (100)	A, B, C
Soil	3/7 (42.8)	A, B	3/3 (100)	B, C
Wastewater	5/6 (83.3)	A, B, C	2/5 (20)	B, C
Water from cooling pads	1/2 (50)	B	1/1 (100)	B
Water in tanks	1/1 (100)	B	0/0	—

Total	15/26 (57.7)		71/79 (89.9)	

## Data Availability

The data used and/or analyzed in this study are available from the corresponding author upon reasonable request.
